# Fecal immunochemical test for colorectal cancer from a prospective cohort with 513,283 individuals: Providing detailed number needed to scope (NNS) before colonoscopy

**DOI:** 10.1097/MD.0000000000004414

**Published:** 2016-09-09

**Authors:** Chien Hua Chen, Chi Pang Wen, Min Kuang Tsai

**Affiliations:** a Digestive Disease Center, Show-Chwan Memorial Hospital, Changhua; b Chung Chou University of Science and Technology, Changhua; c Hungkuang University, Taichung; d Institute of Population Health Sciences, National Health Research Institutes, Zhunan; e China Medical University, Taichung, Taiwan.

**Keywords:** cohort, colorectal cancer, fecal immunochemical test, number needed to scope

## Abstract

The fecal immunochemical test (FIT) is underutilized, in part, because its benefits have not been fully understood. We assessed the relationship of FIT values with cancer incidence and mortality, and explored how repeated administrations of FIT could aid clinicians. A cohort with 513,283 adults in Taiwan participated in a screening program between 1994 and the end of 2007. Colorectal cancer was identified from National Cancer Registry and not from colonoscopy. Positive FIT was FIT ≥ 100 ng/mL. Number needed to scope (NNS) to identify 1 cancer by different FIT values was calculated for the study time. Only 4% of subjects had FIT ≥ 100 ng/mL but contributed 40% of cancer cases, leading to a NNS of 25 for finding 1 in this group. However, within the same FIT ≥ 100 ng/mL, NNS was different by age: 10 for age 60 to 69 years, 42 for age 40 to 49 years, and 156 for age 20 to 39 years. Furthermore, within the same age, NNS was different by FIT values, for instance, 66 for FIT 100 to 199 ng/mL and 12 for FIT 600 to 799 ng/mL, a difference of 5-fold for age 50 to 59 years. The dose–response relationship of FIT can facilitate consultation regarding the need for colonoscopy by providing a quantitative NNS for cancer risk, an index easily understood by patients. Our conclusion made use of (a) age-dependent and (b) quantitative interpretation of FIT values. This single cutpoint practice obliterates a large amount of valuable cancer risk information available to patients.

## Abstract 2

Supplemental Digital Content is available in the text

## 1 Introduction

Colorectal cancer is the third most common cancer worldwide.^[1]^ Several studies have found that screening for colorectal cancer, either by colonoscopy or stool tests, is effective in the average-risk population.^[2,3]^ Colonoscopy, recommended at 10-year intervals for those aged 50 years or older is the most definitive way to identify colorectal cancer,^[4]^ but is limited by the associated cost, the expertise needed to perform the screening, and the potential untoward effects.^[5–8]^ On the other hand, fecal occult blood test (FOBT), known to reduce colorectal cancer mortality by 15% to 33%,^[9–11]^ has been underutilized.^[12,13]^ This underutilization occurred even with the advent of the more acceptable fecal immunochemical test (FIT), which provides quantitative outputs from an automated analysis.^[14–16]^ The reasons for this underutilization were many but varied in different settings. In hospitals, colonoscopy has been the preferred cancer screening mode, while in primary care offices, uncertainties existed for the doctors to interpret specific FIT values to patients whether a referral should be made based on the probability of finding cancers.

The number needed to scope (NNS) is defined as the number of individuals required to detect 1 colorectal cancer by colonoscopy.^[17]^ Patients can easily understand their cancer risk by this quantitative number. Without FIT screening, for the age 50 to 74 years group for example, it had taken 100 colonoscopies to find 1 cancer,^[18]^ but when the cutpoint of 75 or 100 ng/mL was applied with FIT screening, the NNS improved to 12.^[17]^ In a recent randomized control trial in assessing the ability to find cancer between colonoscopy and FIT, NNS was 18 with a positive FIT for age 50 to 69 years, in contrast to 190 when colonoscopy was conducted without FIT screening.^[19]^ Indeed, a surprising finding of this study was that the detection rate for colorectal cancer between the colonoscopy group (without prior FIT) and the FIT group (followed by colonoscopy on positive FIT) was similar.^[19]^ The colonoscopy group, with NNS at 190, actually required 10-fold more colonoscopies than the FIT group to find similar number of cancers. Clearly, the advantages from the use of FIT before colonoscopy in improving the odds of finding cancer were far more than clinicians expected. While there was less-advanced adenoma detected by the FIT group in the trial, which is seen as one weakness of the FIT approach, the authors felt that repeating FIT screening, a recommended practice, not only could identify more advanced adenoma but also more cancer cases, leading to a conclusion favoring FIT eventually. Another important observation of the trial was that FIT screening was far better received by patients than colonoscopy.^[19]^ Nearly one-quarter of those assigned to the colonoscopy group (23%) preferred and opted for FIT screening and not colonoscopy.^[19]^ This preference and higher participation rate for FIT screening is another distinct advantage in promoting FIT before colonoscopy.

The latest data showed that a little over half of US adults aged over 50 years had colonoscopy in the past 10 years as recommended.^[20]^ However, colonoscopy carries considerable costs, special skills, and adverse effects, estimated at 3 to 5 per 1000.^[5–8]^ When the NNS is large, such results should help ease anxiety of the patients and reduce the frequency of colonoscopy needed to achieve meaningful screening.^[21–25]^ Conversely, FIT can provide a compelling reason to proceed with colonoscopy for those subjects reluctant to undergo the test, if a small NNS was found. In most countries like United Kingdom or Taiwan, where screening programs rely mainly on FIT results,^[26,27]^ the age-specific NNS data identified in this study can provide a reference for prioritizing limited resources.

With the availability of more than half a million adults (N = 513,283) with quantitative FIT tests results for each individual, a large sample of FIT-positive subjects (N = 21,353) was identified for characterizing their colorectal cancer risk relative to FIT-negative subjects. One of the objectives of this study was to translate these FIT-positive subjects into age-specific and dose–response NNS. Understanding the characteristics of NNS from FIT results will aid clinicians, along with their patients, in deciding to scope or not to scope, as better utilization of FIT result should improve the cancer detection rate.^[28,29]^

## 2 Methods

### 2.1 Study population

The cohort, consisting of 513,283 adult individuals with age ≥20 years, participated in a standard medical screening program in Taiwan between 1994 and the end of 2007.^[30,31]^ Each participant completed a health history questionnaire with lifestyle and medical history. At the clinic, a battery of laboratory tests was administered, including blood chemistry, blood counts, urinalysis, FIT for stool, functional measurements, and hands-on physicals. Informed consent was obtained, and the study was approved by the Institutional Review Boards at the National Health Research Institutes in Taiwan.

### 2.2 The FIT test

The FIT tool kit was sent to each participant a week before their visit to the clinic with clear instructions described on the kit. The stool was analyzed, and the FIT result was communicated to the participant. Individuals with positive test were advised and referred to outside gastroenterologists for follow-up. This referral was but one of the many referrals made in the counseling session as many abnormal results may have arisen out of the comprehensive screening. We relied on the National Cancer Registry and National death file for identifying colorectal cancer cases and not on colonoscopy, which was not consistently followed after each referral. The program was not specifically colorectal cancer focused, and FIT was part of a battery of tests in a comprehensive screening program. The screening program was responsible for conducting FIT test and communicating its results, but not responsible for making sure it was done right for every step after FIT test.

The automated OC-sensor test (Eiken Chemical Company, Tokyo, Japan), relying on testing antibodies specific for human hemoglobin, was used over the course of the study.^[32]^ The cutpoint for positive values was 100 ng/mL. All numerical values above the cutpoint were recorded and analyzed. The current analysis focused on the initial FIT test at the time of first screening examination, even though nearly half of the cohort had subsequent FIT test at a later date.

### 2.3 Follow-up

The national identification of each cohort participant was matched with the National Death File and National Cancer Registry file. The National Cancer Registry in Taiwan started in 1980, and the National death file started in 1972. The cohort was recruited from 1994 to the end of 2007. The linkage for cancer registry and death file was performed in 2012. As of the end of 2007, a total of 2138 incident cases and 652 deaths with colorectal cancer from the cohort were identified sometime after their FIT test. The National Cancer Registry in Taiwan is a system covers nationwide hospitals, with mandatory reporting when cancer was pathologically established.^[33,34]^

### 2.4 Statistical analysis

The Cox proportional hazard model was used to calculate hazard ratios for cancer incidence and mortality. Ten significant variables were adjusted for in the analysis: age at testing, sex, family history of colorectal cancer, smoking, alcohol, physical inactivity, diabetes, hypertension, anemia, and obesity (Supplementary table S1). Latency time was defined as the duration between initial FIT testing date and the time when each incident cancer was registered or when they died of colorectal cancer.

The 95% confidence interval (CI) for sensitivity, specificity, and NNS (NNS to identify 1 cancer) was calculated by the clinical decision-making calculator.^[35]^ The study focused on the validity of FIT in predicting colorectal cancer (CRC) within the entire follow-up of the cohort. For sensitivity and specificity analysis, we used conventional definition to calculate the false negative by counting those CRC cases within 1 to 2 years after negative FIT, because the CRC screening guideline recommended that FIT be conducted every 1 to 2 years. Beyond 1 to 2 years, a new FIT screening should be conducted, and false negative calculation will then be renewed. NNS is a reciprocal of the positive predictive value (PPV) (NNS = 1/PPV). All statistical tests were 2-sided with the alpha level set at 0.05. All analyses were performed with SAS, version 9.2 (SAS Institute, Inc., Cary, North Carolina).

## 3 Results

With 2138 cancer cases among 513,283 adults for a total of 3793,565 person-years of observation, the incidence rate increased sharply with age, increasing roughly by 5-, 11-, and 25-fold for each successive decade after age 40 years (Table 1). Mortality rate increased with similar trend (Supplementary table S2). Male and those with family history of colorectal cancer had higher incidence rates. Only 4% of the cohort had FIT 100 ng/mL or higher, but contributed 40% of cancer cases.

**Table 1:**
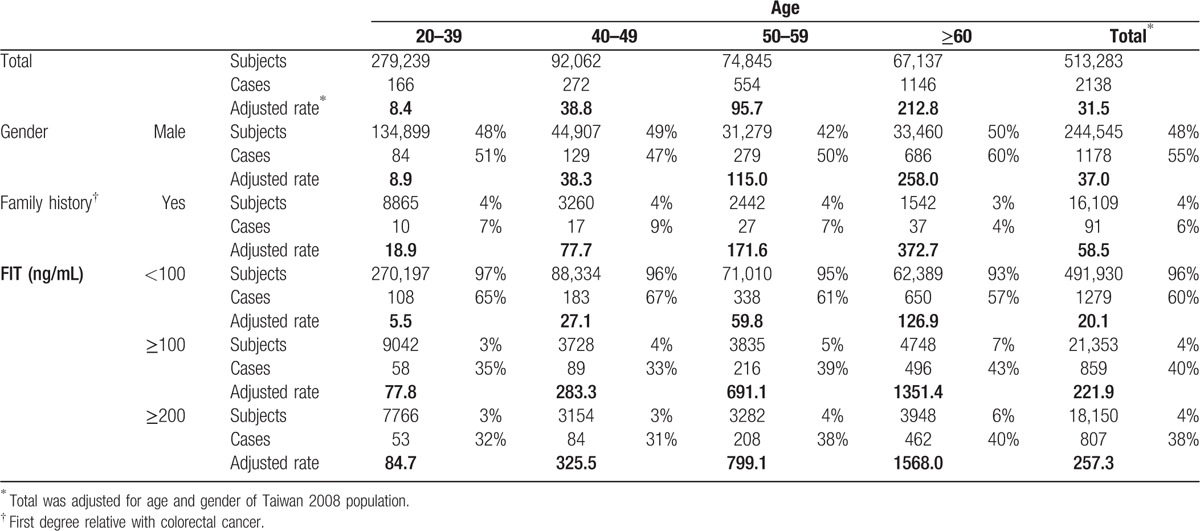
Incidence rate for colorectal cancer by age and by FIT values (per 100,000 person-years).

### 3.1 FIT values and colorectal cancer risk

**Figure 1:**
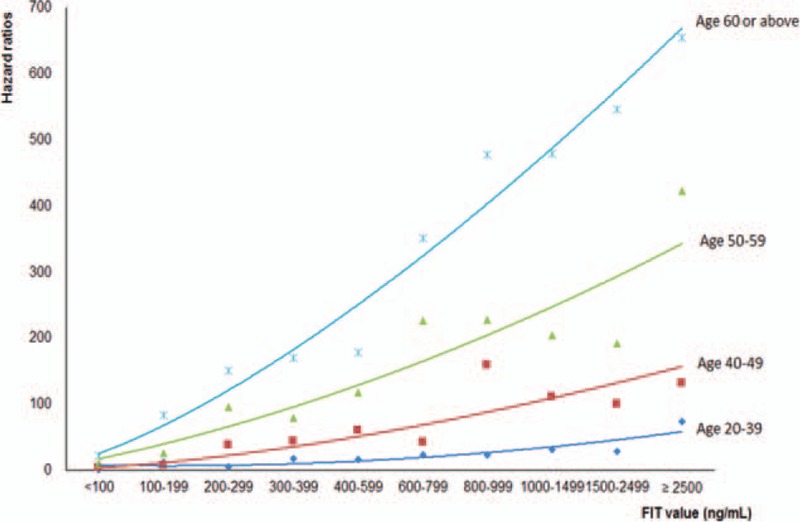
Hazard ratios for colorectal cancer incidence by age groups and by fecal immunochemical test (FIT) values (ng/mL). Reference group: those with age 20 to 39 years with FIT <100 ng/mL. FIT = fecal immunochemical test.

**Figure 2:**
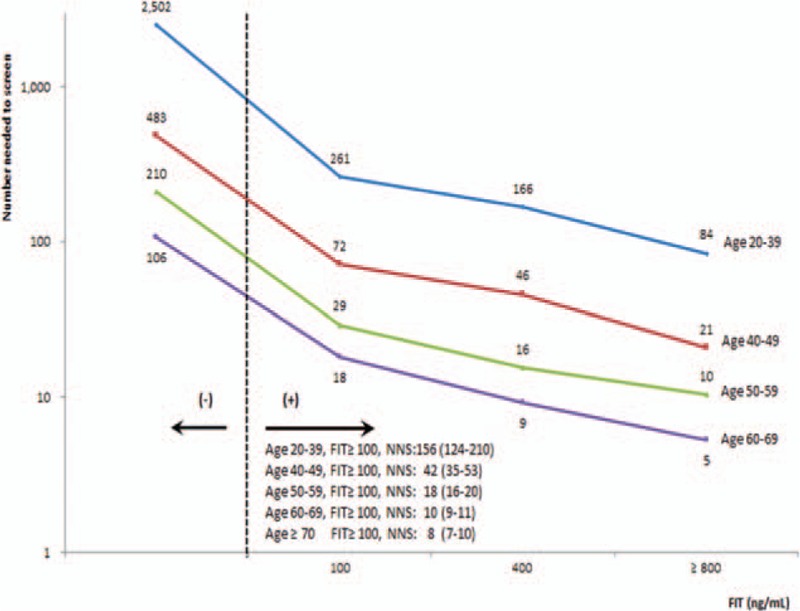
Number of subjects needed to scope in order to identify 1 colorectal cancer by fecal immunochemical test values in different age groups. Age ≥ 70 years had very similar curve as age 60 to 69 years. Point values came from fitted line. FIT = fecal immunochemical test.

Increasing FIT values were associated with increasing colorectal cancer risk in a dose-dependent manner by age (Fig. 1). For instance, compared to subjects with FIT < 100 ng/mL, the risk increased 3.8-, 7.9-, and 22.7-fold for incidence, with similar results for mortality, at FIT values of 100 to 199, 200 to 299, and 800 to 999 ng/mL, respectively (Supplementary figure S1). NNS by different FIT values in 4 different age groups is shown in Fig. 2, stratified by 100 ng/mL. For FIT ≥ 100 ng/mL, NNS varied by age: 10 for age 60 to 69 years, 18 for age 50 to 59 years, 42 for age 40 to 49 years, and 156 for age 20 to 39 years. This dose-dependent relationship was observed across all age groups with older individuals having higher risks and lower NNS with identical FIT values.

### 3.2 Sensitivity and specificity of FIT

The performance of FIT in its ability in detecting or not detecting cancer was evaluated under the way cancer was identified by cancer registry in this study (Table 2), with detection failure defined as those with values <100 ng/mL, presumed to indicate no cancer risk, but found the cancer within the first year (false negative). Both sensitivity (93%) and specificity (96%) calculated this way were high for total cohort. The NNS was smaller for age ≥50 years, 12, than for age <50 years, 87.

**Table 2:**
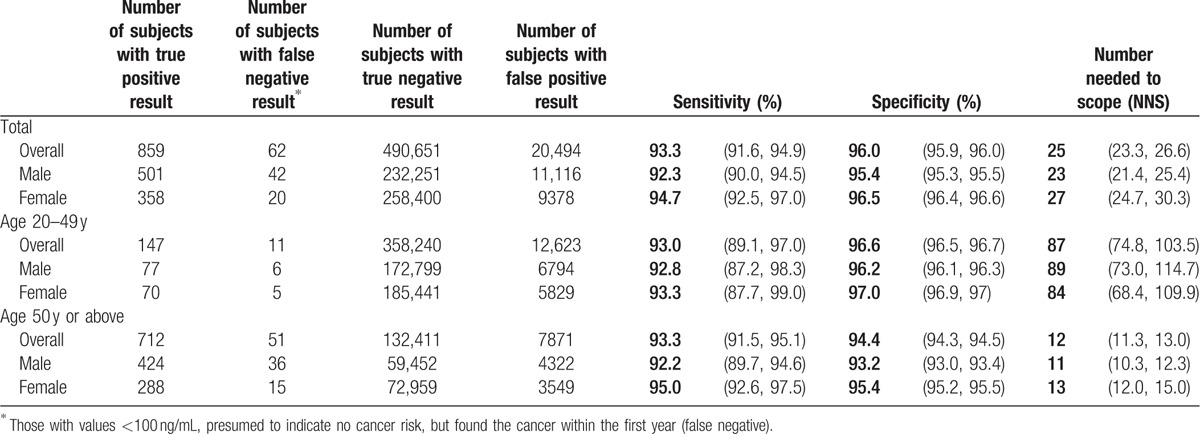
Performance of FIT with 100 ng/mL as threshold to identify colorectal cancer.

### 3.3 FIT values and cancer latency

**Figure 3:**
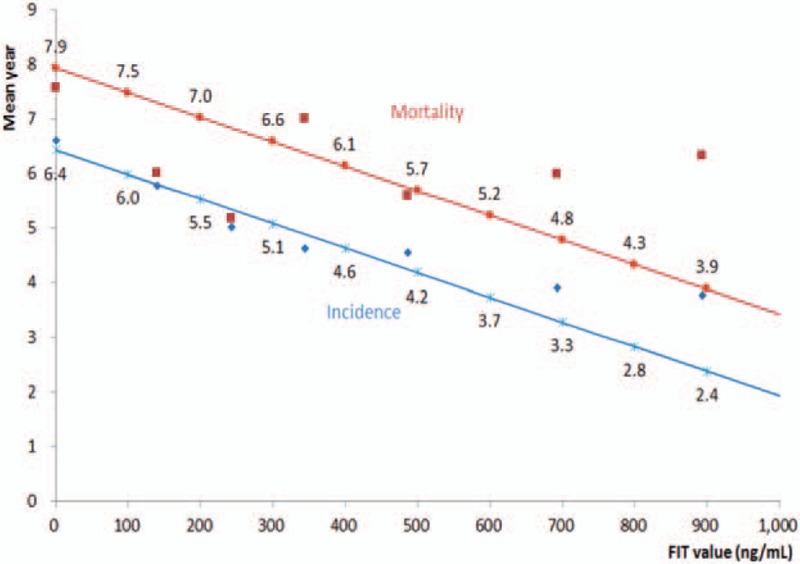
Mean latency between fecal immunochemical test (FIT) screening date and date of colorectal cancer incidence (blue line) or mortality (red line) by FIT values. Incidence: Mean year = 6.4278 − 0.0045 × FIT. FIT = fecal immunochemical test.

There was an inverse relationship between cancer latency (duration between initial FIT test and when cancer was registered) and FIT values, with the larger the FIT value, the shorter the duration for cancer to develop (Fig. 3). For example, with FIT <100 ng/mL, the average latency was 6.6 years, but at 600 to 799 ng/mL, the latency shortened to 3.9 years. By fitting a regression line for all latency time (mean year = 6.4278 − 0.0045 × FIT), we found a linear relationship—up to FIT 1000 ng/mL. For every 200 ng/mL increase in FIT value, cancer developed approximately 1 year sooner.

There were 246 colorectal cancer mortalities in this cohort with initial FIT above 100 ng/mL. Their median/mean FIT values were 773/1276 ng/mL for cancer mortality (Supplementary figure S2). The average time to develop cancer incidence was 3.9 years and to death from cancer was 6.0 years given these median values (Fig. 3).

## 4 Discussion

We identified a relationship between increasing FIT values with increasing colorectal cancer risks that was consistent and striking across all age groups. This dose–response relationship for FIT values above 100 ng/mL, which has never been reported before, was translated into a quantitative cancer index, NNS, or the number of individuals needed to scope for finding 1 cancer. The size of NNS we calculated implied progressive cancer risks, with smaller the NNS, the higher the cancer risk. With this NNS derived from FIT value in hand, doctors are in a better position to discuss with patients the magnitude of cancer risk and the need or urgency in timing for colonoscopy. As FIT has been shown to be far better received than colonoscopy by patients^[13,36]^ and NNS is easily understood, a screening program that integrates FIT screening for colorectal cancer will become much more effective.

FIT value above 100 ng/mL is an often used cutpoint for recommending colonoscopy. However, FIT ≥ 100 ng/mL included a wide range of cancer risks, with NNS between 3 and 576 for all adults (Fig. 2). For age 50 to 59 years in our study, NNS varied between 5 and 66 for different FIT values, a more than 10-fold difference in cancer risk. The current practice of a single cutpoint for a positive FIT overlooked the progressive relationship of risk within this group.^[17,32,37,38]^ Relying on a single cutpoint at 100 ng/mL is to group everyone in the age 50 to 59 years group and assume those above the cutpoint to have identical cancer risk with a single NNS, found at 18 in this study. Our results strongly indicate that this assumption overestimated cancer risk for more than half of this population, particularly for those with FIT below 400 ng/mL. This overestimation could have been avoided and the decision process for follow-up colonoscopy improved if the actual NNS was known and about to be discussed with the patient. On the other hand, 1 recommended practice in the United States is to offer everyone above age 50 years a colonoscopy at 10-year intervals. Approximately, 95% of 50 to 59 years age group in our cohort had a FIT < 100 ng/mL with a corresponding NNS of 191 (95% CI for NNS 170–210). This suggests that both doctors and the patients would hesitate to perform a colonoscopy due to the majority of this age group at that low return level of cancer detection. For those worried well who are concerned of their cancer risk following a FIT measurement at <100 ng/mL, which does exist, it is comforting to know that those who developed cancer later had a median FIT above 700 ng/mL, a level requiring an average of 4 to 6 years to reach for someone with an initial FIT < 100 ng/mL. This warrants monitoring and repeating the FIT measurements annually.^[4,39]^ Given that every 50 ng/mL increase in FIT is associated with 100% increase in cancer risk, monitoring the size of NNS provides ample room for observation and shared decision-making.

With sensitivity at 93%, we found a satisfactory level of performance for FIT in detecting or not detecting colorectal cancer within 1 year at the threshold of 100 ng/mL. This implied that the probability for negative FIT to miss cancer within 1 year was relatively small. Our sensitivity and specificity compared favorably with reported studies when positive FIT was followed by colonoscopy.^[12,17]^

It is of prime importance to note that the NNS identified in this study was for finding colorectal cancer and not just adenoma or advanced adenoma. This is one of the reasons that the NNS in this study, 65 for age 60 to 69 years for example, appeared to be larger than reported NNS.^[18,40]^ Applying an estimated ratio of 6 to 1 between advanced adenoma and cancer, or 18 to 1 for adenoma,^[18]^ the new NNS for adenoma would become comparable to the reported number.^[15,17,18]^ Obviously, if FIT increases above 100 ng/mL, NNS would be reduced by several folds for each of these findings.

Another major finding of this study is the age-dependent nature of FIT. For a given FIT value, cancer risk was not similar across age but varied widely by age. Younger individuals have much larger NNS number than older groups for the same FIT value. For example, given the same FIT at 500 ng/mL, NNS for age 20 to 39 years, 169 (95% CI 91–688), was 10 times the NNS for age 50 to 59 years, 16 (95% CI 11–26), which in turn was nearly twice larger than the number for age 60 to 69 years at 9. We are one of the first to report the increasing cancer risk with increasing FIT values in different age groups. As NNS could vary by age as much as a 15-fold difference for identical FIT, the current practice using a single cutpoint without any age consideration may need modification. For example, those with FIT above 100 ng/mL, a current criteria for positive FIT, NNS varied from 10, 18, 42 to 156 for age 60 to 69, 50 to 59, 40 to 49, and 20 to 39 years, respectively. Thus, for identical FIT values, older people had lower NNS and higher cancer risk, with approximately 5-fold difference between age 40 to 49 years and age ≥60 years.

The finding that higher FIT values were not only associated with higher cancer risk, but also with shortened intervals to develop or to die from colorectal cancer (Fig. 3). For every additional 200 ng/mL increase, cancer latency was roughly shortened by 1 year. This information will aid clinicians in counseling as it bestows a sense of urgency for action directed toward those with higher FIT values or low NNS.

There are important limitations to this study. First, we used cancer registry data as outcome and not the colonoscopy immediately following FIT to identify cancer. The immediate use of colonoscopy will produce more cancer cases than relying on cancer registry to report. Furthermore, while reporting to the National Cancer Registry is legally mandatory, the possibility exists for some cases not registered.^[41]^ Both situations would underestimate the performance of FIT. If FIT can be repeated on an annual basis, the performance would be even better. Second, the entire data came from Asians in Taiwan, and its applicability across races can be questioned. However, when we compared the age-adjusted incidence rate, using US 2000 population as a standard, between Taiwan, 43.1/100,000, and the United States, 43.5/100,000, or age-adjusted mortality rate, 13.8/100,000 for Taiwan, and 15.7/100,000 for the United States,^[42]^ they were remarkably similar, and therefore, the use of same NNS calculated using the Taiwan population to trigger colonoscopy for the US population may not be unreasonable. Third, the cohort comprises paying participants belonging to a relatively higher socioeconomic class (SES) and may not be representative of the national population. However, the cohort was large enough to include all SES classes, and the incidence rate was not noticeably different from national statistics as showed in Supplementary figure S3. Moreover, the relative risks were internally standardized and not subject to confounding due to SES variation. With most of the cohort representing an average-risk population, this study is different from most other reports evaluating high-risk populations.^[32,37,38]^ Thus, our data provides findings that are more applicable to the general public.

In summary, our study examined the predictive power of initial FIT for colorectal cancer risk from a large prospective cohort. We uncovered a dose–response relationship of colorectal cancer with FIT for each age group, highlighting the importance of age on interpreting a single cutpoint or a quantitative value from FIT.

## Supplementary Material

Supplemental Digital Content
