# Supplementary material for: Fecal immunochemical test for colorectal cancer from a prospective cohort with 513,283 individuals: Providing detailed number needed to scope (NNS) before colonoscopy
Source: Medicine (Baltimore). 2016 Sep 9;95(36):e4414. doi: 10.1097/MD.0000000000004414 (PMC5023859; doi:10.1097/MD.0000000000004414)
Supplement: Supplemental Digital Content [file medi-95-e4414-s001.doc]

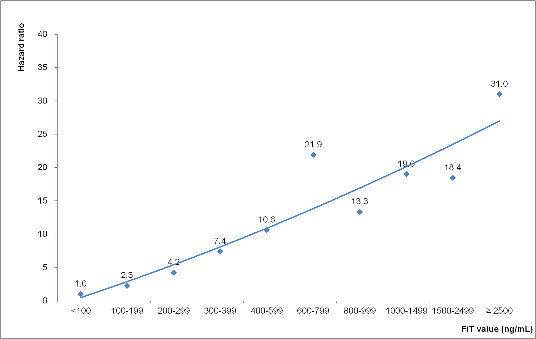

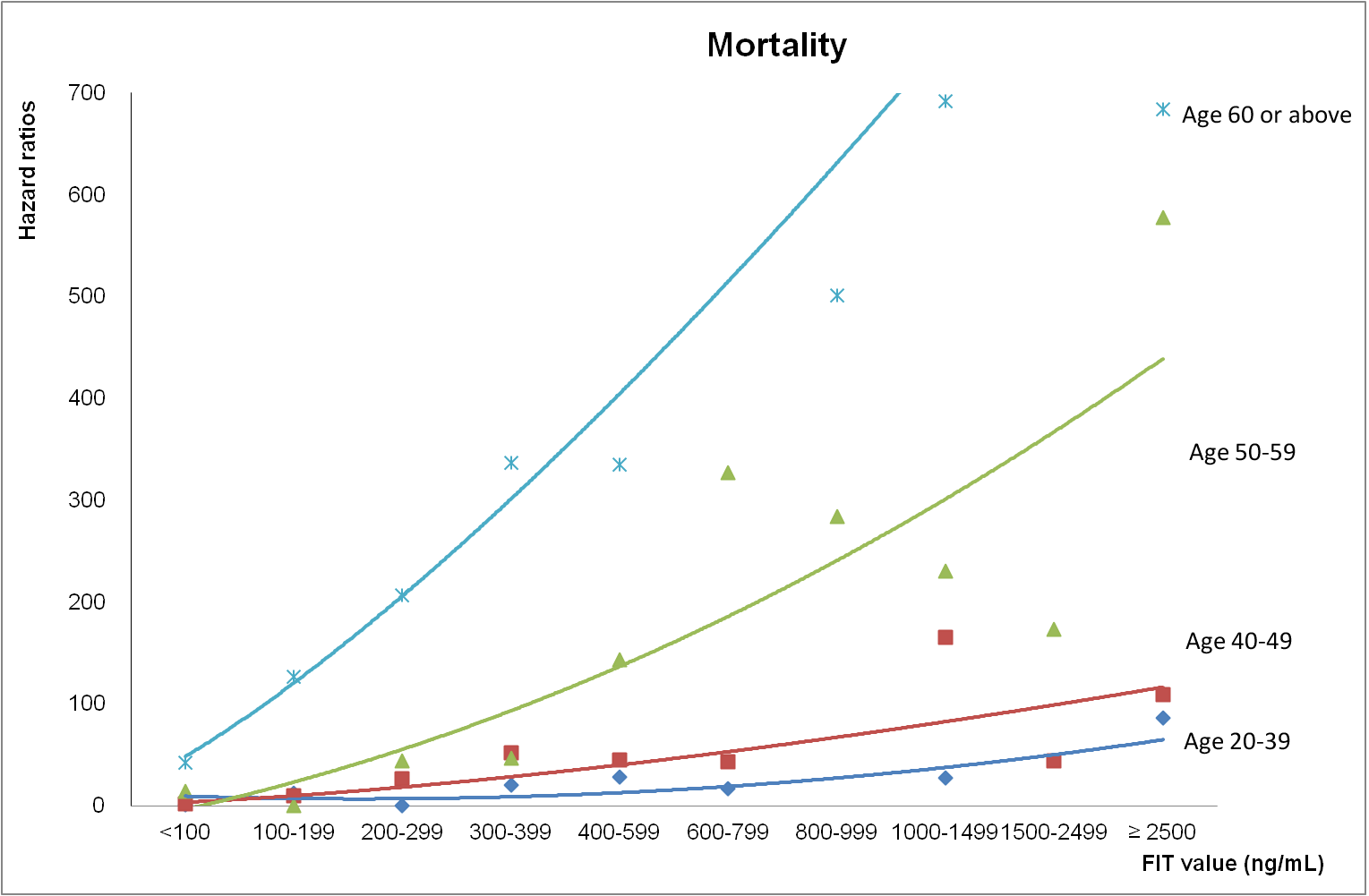


**Supplementary Figure S1. Hazard ratios for colorectal cancer mortality by age groups and by FIT values (ng/mL)**

Reference group: those with age 20-39 years and FIT <100 ng/mL

FIT: (Fecal immunochemical test)


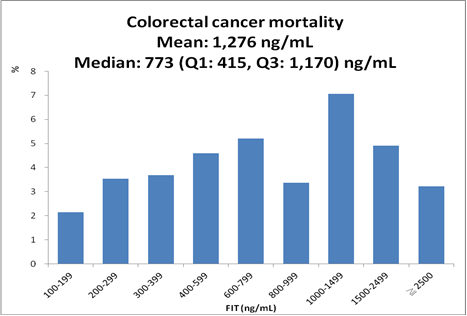


**Supplementary figure S2. Distribution of colon cancer death by FIT values among those with FIT test above 100 ng/mL**

FIT: (Fecal immunochemical test)


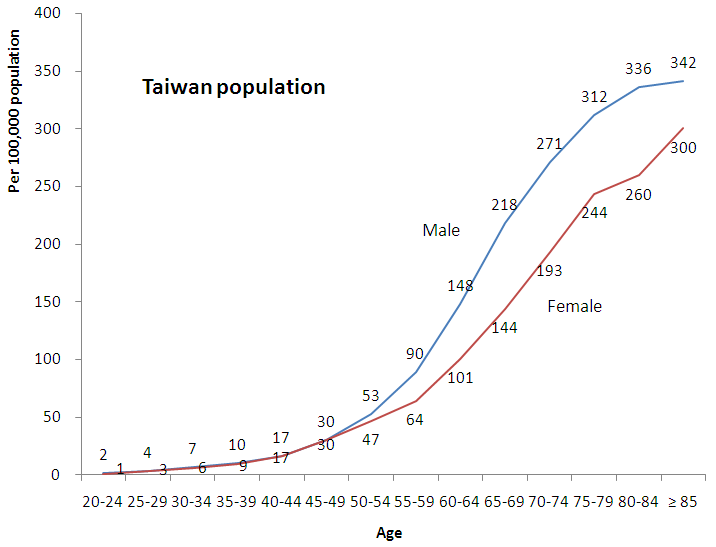

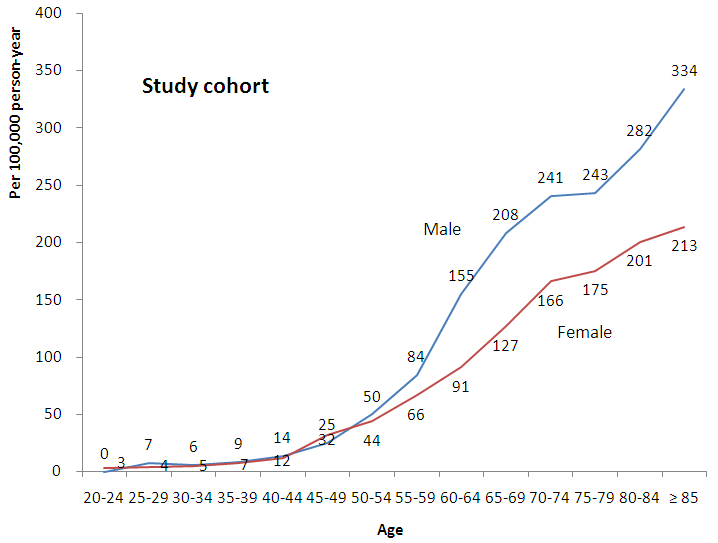


**Supplementary figure S3. Age specific colorectal incidence by gender for Taiwan (2001~2005) and for study cohort (1994-2007) per 100,000**

| **Supplementary table S1. Incidence rate for colorectal cancer by age and by risk factors (per 100,000 person-year)** | | | | | | | | | | | | | | | | | | |
| --- | --- | --- | --- | --- | --- | --- | --- | --- | --- | --- | --- | --- | --- | --- | --- | --- | --- | --- |
|  |  |  | Age | | | | | | | | | | | | | | | |
|  |  |  | **20-39** | | | **40-49** | | |  | **50-59** | | | ≥ **60** | | | Total‡‡ | | |
| Total |  | Subjects | 279,239 |  |  | 92,062 |  |  |  | 74,845 |  |  | 67,137 |  |  | 513,283 |  |  |
|  | | Cases | 166 |  |  | 272 |  |  |  | 554 |  |  | 1,146 |  |  | 2,138 |  |  |
|  |  | Adjusted rate | **8.4** |  |  | **38.8** |  |  |  | **95.7** |  |  | **212.8** |  |  | **31.5** |  |  |
| Gender | Male | Subjects | 134,899 |  | 48% | 44,907 |  | 49% |  | 31,279 |  | 42% | 33,460 |  | 50% | 244,545 |  | 48% |
|  |  | Cases | 84 |  | 51% | 129 |  | 47% |  | 279 |  | 50% | 686 |  | 60% | 1,178 |  | 55% |
|  |  | Adjusted rate | **8.9** |  |  | **38.3** |  |  |  | **115.0** | ***** |  | **258.0** | ***** |  | **37.0** | ***** |  |
| Family history† | Yes | Subjects | 8,865 |  | 4% | 3,260 |  | 4% |  | 2,442 |  | 4% | 1,542 |  | 3% | 16,109 |  | 4% |
|  |  | Cases | 10 |  | 7% | 17 |  | 9% |  | 27 |  | 7% | 37 |  | 4% | 91 |  | 6% |
|  |  | Adjusted rate | **18.9** | ***** |  | **77.7** | ***** |  |  | **171.6** | ***** |  | **372.7** | ***** |  | **58.5** | ***** |  |
| Smoking‡ | Yes | Subjects | 68,241 |  | 30% | 22,758 |  | 31% |  | 14,891 |  | 26% | 15,715 |  | 32% | 121,605 |  | 30% |
|  |  | Cases | 47 |  | 37% | 58 |  | 33% |  | 121 |  | 34% | 317 |  | 41% | 543 |  | 38% |
|  |  | Adjusted rate | **10.2** |  |  | **37.9** |  |  |  | **112.8** | ***** |  | **268.5** | ***** |  | **40.3** | ***** |  |
| Drinking§ | Yes | Subjects | 43,463 |  | 19% | 20,158 |  | 28% |  | 14,516 |  | 26% | 12,204 |  | 25% | 90,341 |  | 22% |
|  |  | Cases | 30 |  | 23% | 62 |  | 35% |  | 122 |  | 34% | 251 |  | 33% | 465 |  | 33% |
|  |  | Adjusted rate | **9.2** |  |  | **40.4** |  |  |  | **113.3** | ***** |  | **273.0** | ***** |  | **39.0** | ***** |  |
| Physical inactivity | Inactive¶ | Subjects | 147,452 |  | 59% | 40,836 |  | 52% |  | 30,434 |  | 49% | 22,556 |  | 43% | 241,278 |  | 55% |
|  |  | Cases | 79 |  | 60% | 108 |  | 56% |  | 214 |  | 53% | 328 |  | 40% | 729 |  | 47% |
|  |  | Adjusted rate | **8.1** |  |  | **38.1** |  |  |  | **97.8** |  |  | **197.2** |  |  | **32.4** |  |  |
| Diabetes|| | Yes | Subjects | 2,955 |  | 1.1% | 4,354 |  | 5% |  | 8,337 |  | 11% | 10,804 |  | 16% | 26,450 |  | 5% |
|  |  | Cases | 2 |  | 1.2% | 15 |  | 6% |  | 70 |  | 13% | 197 |  | 17% | 284 |  | 13% |
|  |  | Adjusted rate | **10.8** |  |  | **47.5** |  |  |  | **117.1** | ***** |  | **256.8** | ***** |  | **39.8** | ***** |  |
| Hypertension** | Yes | Subjects | 14,148 |  | 5% | 14,034 |  | 15% |  | 25,480 |  | 34% | 37,056 |  | 55% | 90,718 |  | 18% |
|  |  | Cases | 11 |  | 7% | 42 |  | 15% |  | 223 |  | 40% | 679 |  | 59% | 955 |  | 45% |
|  |  | Adjusted rate | **11.1** |  |  | **39.2** |  |  |  | **112.8** | ***** |  | **229.9** | ***** |  | **35.4** | ***** |  |
| Anemia†† | Yes | Subjects | 20,547 |  | 7% | 9,588 |  | 10% |  | 6,134 |  | 8% | 8,759 |  | 13% | 45,028 |  | 9% |
|  |  | Cases | 24 |  | 14% | 36 |  | 13% |  | 61 |  | 11% | 199 |  | 17% | 320 |  | 15% |
|  |  | Adjusted rate | **15.5** | * |  | **47.5** | * |  |  | **121.7** | * |  | **287.8** | * |  | **42.3** | * |  |
| Obesity | BMI≥ 30 | Subjects | 8,447 |  | 3% | 4,018 |  | 4% |  | 4,061 |  | 5% | 3,382 |  | 5% | 19,908 |  | 4% |
|  |  | Cases | 5 |  | 3% | 21 |  | 8% |  | 40 |  | 7% | 51 |  | 4% | 117 |  | 5% |
|  |  | Adjusted rate | **9.2** | ***** |  | **67.9** | ***** |  |  | **123.5** | ***** |  | **193.1** |  |  | **36.1** | ***** |  |
| * Significant increase (p<0.05) by examining hazard ratios for colorectal cancer incidence when comparing within each risk factor  † First degree relative with colorectal cancer  ‡ Current smoker  § Regular drinker drinking 3 cups or above per week and more than 2 drinks each time  ¶ Inactive is no exercise or less than 3.75 MET-hour per week (less than 1 hour per week)  || Diabetes is fasting glucose ≥ 126 mg/dL or on medication or self-reported history  ** Hypertension is systolic blood pressure ≥ 140 mmHg or on medication on self-report history  †† Anemia is hemoglobin <13 gm/dL for men or <12 gm/dL for women  ‡‡ Adjusted for age and gender of Taiwan 2008 population | | | | | | | | | | | | | | | | | | |

| **Supplementary table S2. Mortality rate for colorectal cancer by age and by fecal immunochemical test**  **FIT values (per 100,000 person-year)** | | | | | | | | | | | | | | | | | |
| --- | --- | --- | --- | --- | --- | --- | --- | --- | --- | --- | --- | --- | --- | --- | --- | --- | --- |
|  |  |  | Age | | | | | | | | | | | |  |  |  |
|  |  |  | 20-39 | | | 40-49 | | | 50-59 | | | ≥ 60 | | | Total* | | |
| Total |  | Subjects | 279,239 |  |  | 92,062 |  |  | 74,845 |  |  | 67,137 |  |  | 513,283 |  |  |
|  | | Death | 44 |  |  | 51 |  |  | 146 |  |  | 411 |  |  | 652 |  |  |
|  |  | Adjusted rate | **1.9** |  |  | **6.4** |  |  | **22.0** |  |  | **68.7** |  |  | **8.9** |  |  |
| FIT  (ng/mL) | <100 | Subjects | 270,197 |  | 97% | 88,334 |  | 96% | 71,010 |  | 95% | 62,389 |  | 93% | 491,930 |  | 96% |
|  |  | Death | 30 |  | 70% | 34 |  | 67% | 94 |  | 64% | 248 |  | 60% | 406 |  | 62% |
|  |  | Adjusted rate | **1.4** |  |  | **4.5** |  |  | **15.1** |  |  | **44.6** |  |  | **6.1** |  |  |
|  | ≥ 100 | Subjects | 9,042 |  | 3% | 3,728 |  | 4% | 3,835 |  | 5% | 4,748 |  | 7% | 21,353 |  | 4% |
|  |  | Death | 13 |  | 30% | 17 |  | 33% | 52 |  | 36% | 164 |  | 40% | 246 |  | 38% |
|  |  | Adjusted rate | **15.9** |  |  | **48.0** |  |  | **144.0** |  |  | **385.5** |  |  | **52.7** |  |  |
|  | ≥ 200 | Subjects | 7,766 |  | 3% | 3,154 |  | 3% | 3,282 |  | 4% | 3,948 |  | 6% | 18,150 |  | 4% |
|  |  | Death | 11 |  | 25% | 17 |  | 33% | 51 |  | 35% | 153 |  | 37% | 232 |  | 36% |
|  |  | Adjusted rate | **16.1** |  |  | **58.3** |  |  | **168.7** |  |  | **442.4** |  |  | **61.3** |  |  |
| * Rate for total was adjusted to age and gender of Taiwan 2008 population | | | | | | | | | | | | | | | | | |
